# Case Report: A rare case of EBV-positive inflammatory follicular dendritic cell sarcoma occurring in the testes

**DOI:** 10.3389/fonc.2026.1723376

**Published:** 2026-04-28

**Authors:** Rongrong Meng, Xuefen Wang, Zhengzheng Shi

**Affiliations:** Department of Pathology, Ningbo Hospital of Integrated Traditional Chinese and Western Medicine, Ningbo, Zhejiang, China

**Keywords:** EBV-positive inflammatory, Epstein-Barr virus, follicular dendritic cell sarcoma, immunohistochemistry, testes

## Abstract

Follicular dendritic cell sarcoma (FDCS) is an exceedingly rare low-grade malignant tumor originating from follicular dendritic cells. It is classified into two subtypes: the classic type and the EBV-positive inflammatory follicular dendritic cell sarcoma (EBV+ iFDCS). While EBV+ iFDCS predominantly arises in abdominal organs such as the liver and spleen, its morphological spectrum is broad, rendering diagnosis challenging. Here, we report an exceptionally rare case of EBV+ iFDCS occurring in the testes, a site previously unreported in the literature.

## 1 Introduction

Follicular Dendritic Cells (FDCs) are antigen-presenting cells primarily distributed in the white pulp of the spleen, lymph nodes, and intestinal lymphoid tissues. Follicular Dendritic Cell Sarcoma (FDCS) is a low-grade malignant tumor originating from FDCs. According to the 2022 World Health Organization(WHO) Classification of Haematolymphoid Tumours (5th edition), FDCS is mainly classified into the classic type and the EBV-positive inflammatory type (EBV-positive inflammatory follicular dendritic cell sarcoma, EBV+ iFDCS) (1). Research has shown that the SSTR2 gene can help distinguish between these subtypes, with the classic type typically being SSTR2 positive and the EBV-positive inflammatory type usually negative (2).

EBV-positive inflammatory follicular dendritic cell sarcoma (EBV+ iFDCS) is rare, primarily occurring in the liver and spleen (3, 4), with a few cases reported in the intestines, lungs, pancreas, and adrenal glands (5–7). The median age of onset is 54.5 years (range: 19–88 years), with a higher incidence in females (male-to-female ratio of 1.7:1) (8). Clinical symptoms are usually nonspecific, mainly presenting as abdominal discomfort, bloating, or pain.

The pathogenesis of EBV+ iFDCS remains unclear, but most scholars believe it is related to Epstein-Barr Virus (EBV) infection (9). The 2022 WHO classification establishes that the detection of EBER within tumor cells is essential for the diagnosis of EBV+ iFDCS (1). However, previous literature has reported two cases of EBV-negative inflammatory pseudotumor (IPT)-like FDCS in the intestines (10, 11), which, according to the current classification, would require re-evaluation and likely fall into a different diagnostic category.

This historical context underscores the critical importance of EBER testing in the diagnostic algorithm. This case represents the first reported instance of EBV+ iFDCS occurring in the testes, highlighting the importance of considering this rare tumor in the differential diagnosis of testicular masses.

## 2 Case report

### 2.1 Clinical history and ultrasound findings

A 61-year-old male presented to our hospital primarily due to left scrotal discomfort in April 2023. He had no significant past medical history of chronic diseases, no previous testicular trauma or infections, and no family history of testicular or hematolymphoid malignancies. There was no history of smoking or alcohol abuse. HIV serology was negative. The patient had no history of organ transplantation, autoimmune diseases, or long-term use of corticosteroids or immunosuppressants that could lead to immunosuppression. He reported left scrotal swelling and discomfort, but no systemic symptoms such as fever or weight loss were observed.

**Figure 1:**
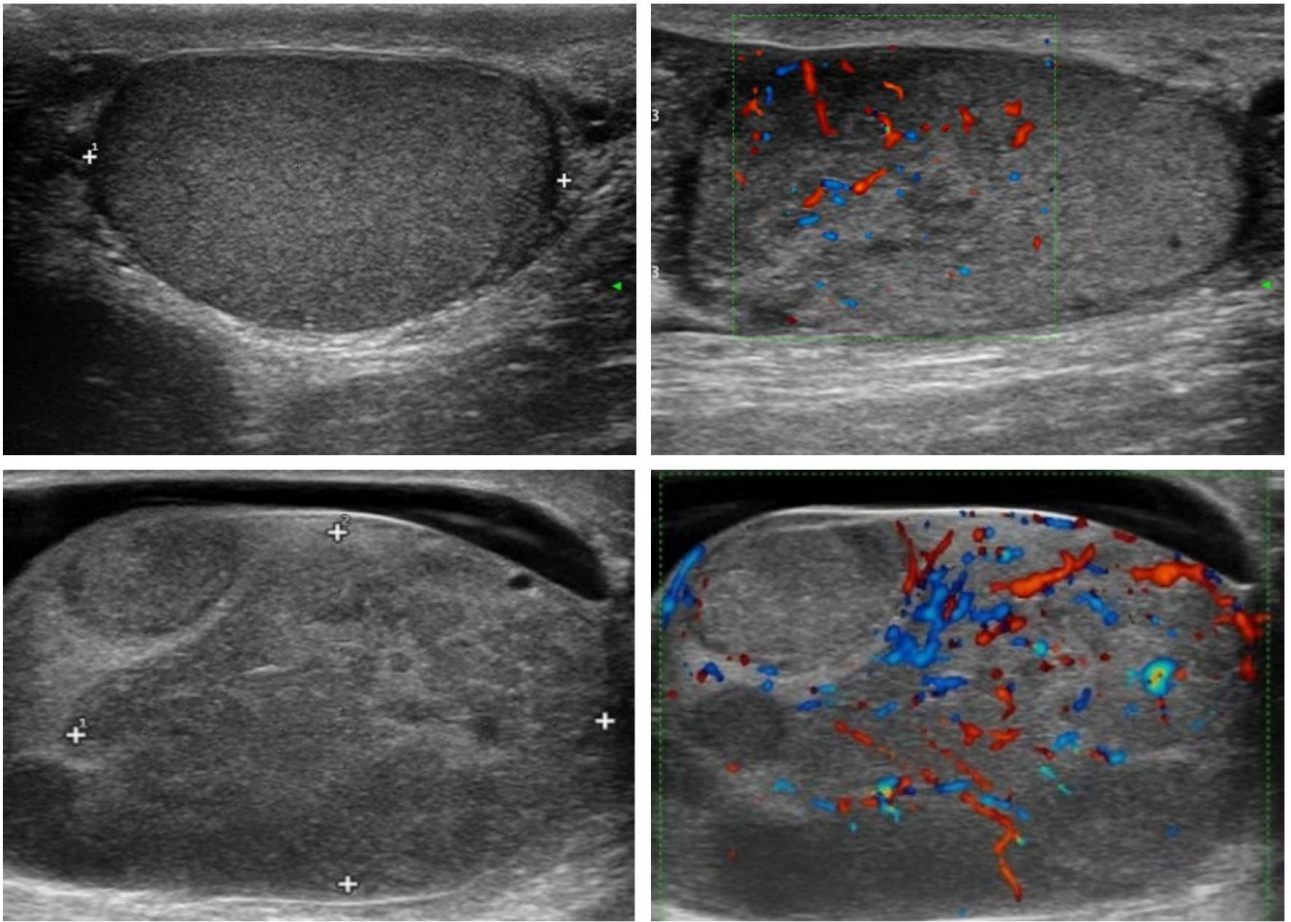
Testicular ultrasound and color Doppler flow imaging findings. Ultrasound and color Doppler flow imaging (CDFI) images are shown in a 2 × 2 panel, arranged from left to right and top to bottom. Ultrasound and color Doppler flow imaging (CDFI) of the testis at the patient’s initial visit. Follow-up ultrasound and CDFI images showing heterogeneous internal echogenicity with multiple hypoechoic areas, the largest measuring approximately 39 × 30 mm, with abundant internal blood flow signals. (2×2 grid): A (top-left), B (top-right), C (bottom-left), D (bottom-right).

Initial ultrasound revealed a left testis measuring 42 × 27 × 25 mm, with a complete capsule, a less smooth surface, and uneven internal echogenicity. No obvious space-occupying lesion was detected, but Color Doppler Flow Imaging (CDFI) showed internal blood flow signals, suggesting inflammation (**Figures 1A, B**).

Anti-inflammatory treatment was recommended. In December 2023, the patient noted a significant enlargement of the left scrotal mass. Repeat ultrasound showed the left testis had enlarged to 50 × 40 × 34 mm, with a complete capsule, a less smooth surface, and multiple hypoechoic areas. CDFI demonstrated abundant blood flow signals, and a 14 mm fluid-filled dark zone was observed in the sheath cavity, indicating effusion (**Figures 1C, D**). These findings raised suspicion of a neoplastic lesion, prompting surgical removal of the left testis and epididymis. The ultrasound and color Doppler flow imaging findings are shown in **Figure 1**, and the timeline of clinical presentation, diagnostic workup, and disease course is summarized in **Table 1**.

**Table 1:** Timeline of clinical presentation, diagnostic workup, and disease course.

Date	Clinical event or examination	Main findings	Intervention
April 2023	Initial presentation	Left scrotal discomfort, no systemic symptoms	Clinical observation
April 2023	Initial Ultrasound	Testis 42×27×25mm, uneven echogenicity, sparse blood flow	Anti-inflammatory treatment recommended
April-December 2023	Clinical course	Slight symptom relief after anti-inflammatory treatment, but gradual mass enlargement	Outpatient follow-up
December 2023	Follow-up Ultrasound	Testis enlarged to 50×40×34mm, abundant blood flow, hydrocele	Surgery recommended
December 2023	Surgical intervention	Left orchiectomy and epididymectomy performed	
Postoperative	Pathological diagnosis	EBV+iFDCS confirmed	
Post-discharge	Further management	Referred to a higher-level hospital	Patient lost to follow-up

### 2.2 Diagnostic methods

#### 2.2.1 Laboratory examination

Preoperative laboratory examination (December 2023) showed that all complete blood count parameters were within normal ranges: white blood cell count was 5.8×10^9^/L (reference range: 3.5-9.5), neutrophil percentage was 58.7% (reference range: 40.0-75.0%). High-sensitivity C-reactive protein was 6.2 mg/L (reference range: 0–10 mg/L), showing no significant elevation. HIV was negative.

#### 2.2.2 Pathological examination

Gross examination revealed a grey-white soft mass measuring 55 × 40 × 28 mm within the testis, which measured 60 × 50 × 40 mm. The mass had a complete capsule and clear borders, with areas of partial necrosis, with necrosis accounting for approximately 30% of the total tumor volume. The epididymis measured 40 × 10 × 6 mm, and the spermatic cord was 140 mm in length and 25 mm in diameter.

**Figure 2:**
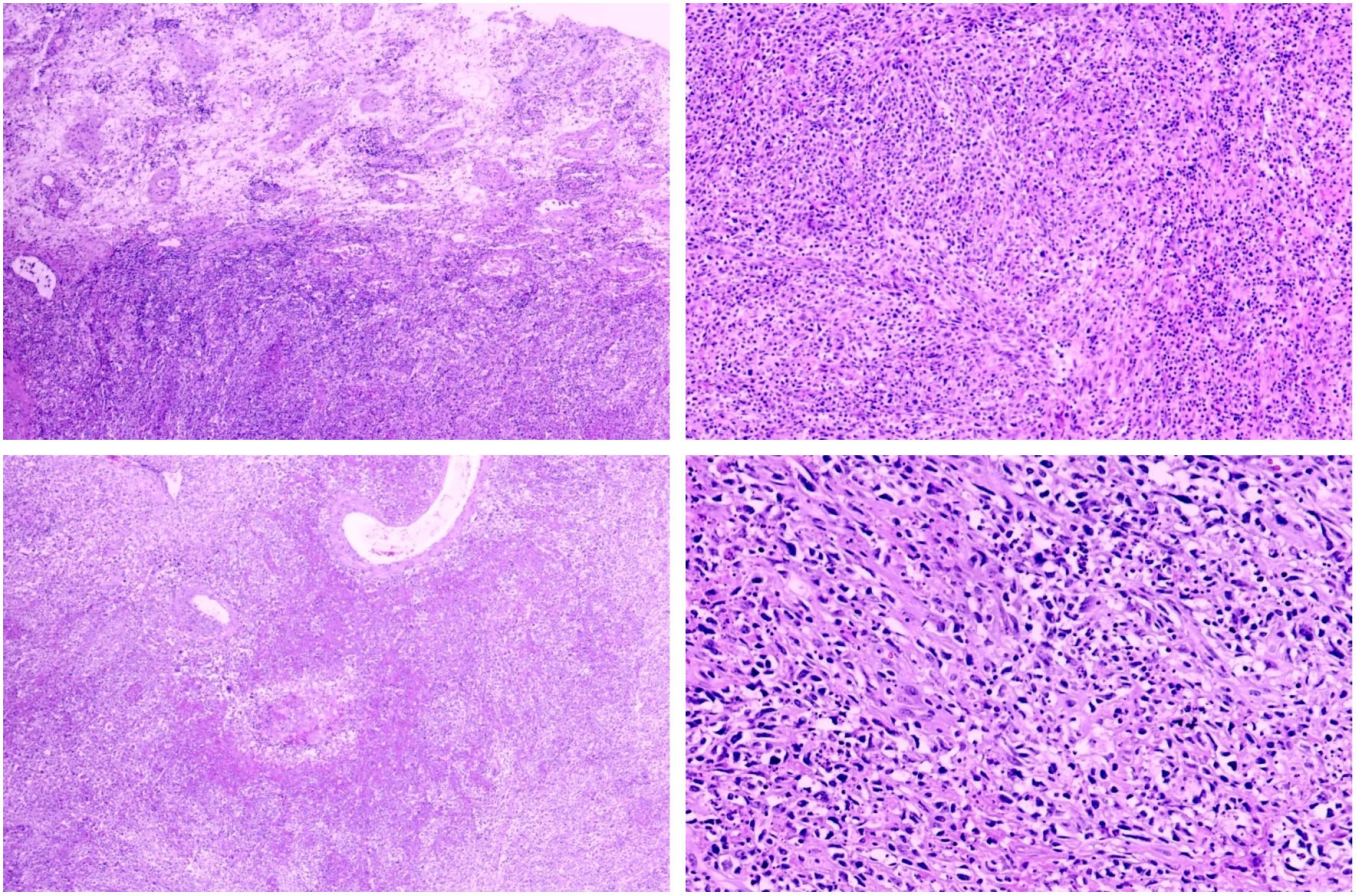
Histopathological features of the tumor. Representative hematoxylin and eosin (H&E)-stained sections are shown in a 2 × 2 panel, arranged from left to right and top to bottom. Diffuse invasive growth with clear boundaries from surrounding normal tissue. Spindle and oval tumor cells arranged in whorls or bundles, with a background of lymphocytes, plasma cells, and neutrophils. Central necrosis with extensive neutrophil infiltration. Tumor cells with light red cytoplasm, irregular nuclei, and clear nuclear membranes. A (top-left), B (top-right), C (bottom-left), D (bottom-right).

Histopathological analysis of hematoxylin and eosin (H&E)-stained sections showed diffuse invasive growth of tumor tissue with significant necrosis, though the boundary with surrounding normal testicular tissue remained clear (**Figure 2A**). Tumor cells were predominantly spindle-shaped and oval, arranged in whorls or bundles, with a background of lymphocytes, plasma cells, and neutrophils (**Figure 2B**). Necrotic areas exhibited extensive neutrophil infiltration (**Figure 2C**). Tumor cells displayed light red cytoplasm, spindle-shaped or oval nuclei with clear nuclear membranes, irregular nuclear contours, and small purple-stained nucleoli (**Figure 2D**). Mitotic figures were observed, and vascular proliferation was evident, with some vessels exhibiting a hyalinized appearance. The histopathological features are shown in **Figure 2**.

#### 2.2.3 Immunohistochemistry, *in situ* hybridization, and molecular testing

**Figure 3:**
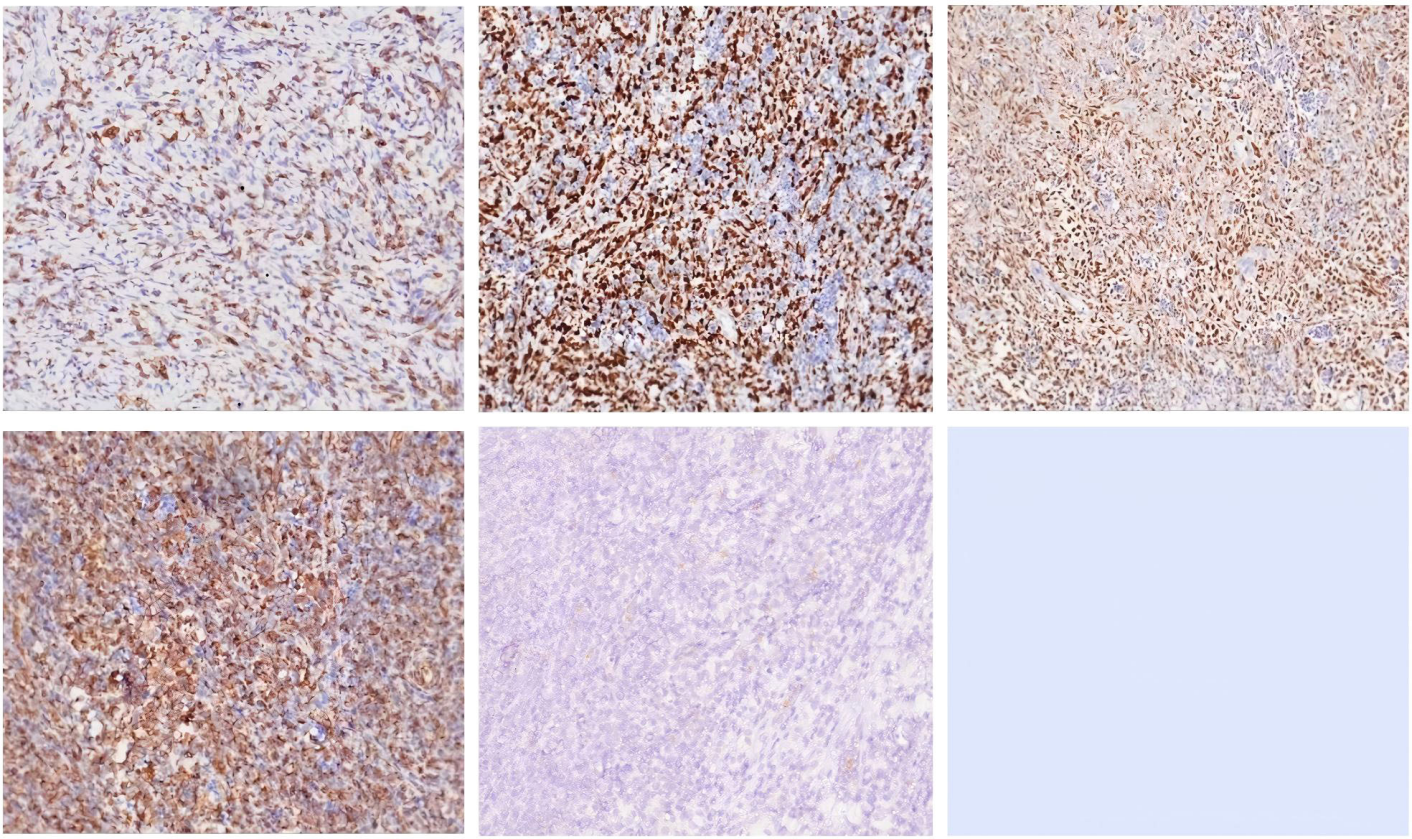
Immunohistochemical staining results. Immunohistochemical staining images are shown in an irregular 3 × 2 panel, with three images in the top row and two images in the bottom row (the bottom right position is left blank). The panels are arranged from left to right and top to bottom. Strong CD3 positivity in background cells. High Ki-67 proliferation index (hotspot area ~60%). Diffuse EBER positivity in tumor cells. Strong vimentin expression in tumor cells. Focal CD35 positivity in tumor cells. (irregular 3×2 layout; top row: 3 images, bottom row: 2 images, bottom-right. blank): A (top-left), B (top-middle), C (top-right), D (bottom-left), E (bottom-middle). The blank position has no label.

The results of immunohistochemistry, *in situ* hybridization, and molecular testing are summarized in **Table 2**. Representative immunohistochemical staining images are shown in **Figure 3**.

**Table 2:** Comprehensive immunohistochemical, *in situ* hybridization, and molecular detection results.

Category	Markers	Results	Notes
*In Situ* Hybridization	EBER	Diffuse positive	**Figure 3C**
IHC Positive Markers	Vimentin	Strong diffuse positive	**Figure 3D**
	CD35	Focal positive	**Figure 3E**
IHC negative markers			
• Follicular dendritic cell markers	CD21, CD23, D2-40	Negative	
• Germ cell tumor markers	PLAP, SALL4, OCT4, CD117	Negative	
• Melanocytic markers	SOX10, HMB45, S-100	Negative	
• Lymphocyte markers	TIA-1, GranzymeB, CD56	Negative	
• Mesenchymal markers	ALK, Calponin	Negative	
• Histiocytic markers	CD1α, CD68, CD163	Negative	
• Myogenic markers	SMA, Desmin	Negative	
• Epithelial markers	CK(Pan), EMA	Negative	
Background Cell Markers	CD3	Strong positive	**Figure 3A**
Proliferation Index	Ki-67	~60% (hotspot areas)	**Figure 3B**
Molecular Test	TCR gene rearrangement	Negative	

To further clarify the nature of the tumor, Epstein-Barr virus-encoded small RNA (EBER) *in situ* hybridization was performed to assess EBV infection status. Detection was carried out on formalin-fixed, paraffin-embedded tumor tissue sections obtained after surgical resection, and the results showed diffuse strong positive signals within tumor cell nuclei.

It should be specifically noted that this study only performed tissue-level EBER testing and did not perform peripheral blood EBV DNA quantitative PCR testing. Therefore, data on the patient’s systemic EBV viral load are lacking, and it is not possible to assess the presence of active viral replication or prior infection history. This is one of the main limitations of this study.

### 2.3 Patient and disease course evolution

The patient underwent left radical orchiectomy in December 2023 and was discharged after uneventful postoperative recovery. Subsequently, the patient was referred to a higher-level hospital for further evaluation and management.

However, due to personal health considerations and other multifaceted reasons, the patient declined long-term follow-up and further management at our institution. Despite multiple attempts to contact him for surveillance, no clinical or imaging data beyond the immediate postoperative period are available. The patient provided written informed consent for the use of his clinical and pathological data for this academic case report and publication, but long-term survival data and disease progression status remain unavailable.

## 3 Discussion

### 3.1 Pathological features and diagnostic challenges

EBV+iFDCS exhibits significant heterogeneity in the expression of FDC markers, ranging from diffuse positivity, focal positivity, to complete negativity (12, 13). In the present case, CD21, CD23, and D2–40 were all negative, with only CD35 showing focal positivity. This variable expression of FDC markers is an important characteristic of EBV+iFDCS, not a diagnostic obstacle. Given the heterogeneity of FDC marker expression, EBER *in situ* hybridization plays a central role in diagnosis.

The focal positive expression of CD35 has important value in the diagnosis of EBV+iFDCS. As a complement receptor, CD35 is expressed earlier in the differentiation process of follicular dendritic cells, while the expression of CD21 and CD23 is associated with cell maturity. The pattern of CD35 focal positivity with CD21/CD23 negativity observed in this case reflects the tumor cells being in a specific differentiation state, which is consistent with the “abnormal immunophenotype” commonly seen in EBV+iFDCS.

SSTR2 is an important marker in the differential diagnosis of FDCS. Studies have shown that SSTR2 is mostly positive in classic FDCS, while it is usually negative in EBV+iFDCS. SSTR2 testing could not be performed in this case due to technical reasons, which is a limitation of this study.

EBV plays a crucial role in the pathogenesis of EBV+iFDCS. Research indicates that EBV exhibits a clonal phenotype in the tumor, suggesting that viral infection occurs before cellular transformation and replicates with the clonal expansion of tumor cells. EBER and LMP-1 proteins expressed during EBV latent infection can enhance the CD40 signaling pathway, inhibiting apoptosis of follicular dendritic cells and promoting their proliferation. Genomic studies have also revealed that EBV+iFDCS has unique molecular features, involving somatic mutations in multiple key signaling pathways including NF-κB signaling, RTK/RAS/PI(3)K, and Hippo pathways.

Additionally, EBV+iFDCS may express other markers including vimentin, EMA, S100, SMA, and ER (14). In this case, vimentin expression was strongly positive, further supporting a mesenchymal origin.

### 3.2 Differential diagnosis

The diagnosis of EBV+ iFDCS in this case presented a significant challenge due to the incomplete immunophenotypic profile. The classic FDCS markers CD21 and CD23 were negative, with only focal positivity for CD35. Furthermore, while diffuse EBER positivity is a prerequisite for the diagnosis, it is not entirely specific and can rarely be seen in other entities, such as classical inflammatory pseudotumor.

Therefore, the diagnosis relied on a combination of findings, effectively making it a diagnosis of exclusion based on a comprehensive diagnostic workup. As detailed in **Table 3**, the diagnosis is supported by three key pillars: (1) Compatible morphological features (spindle cells arranged in whorls or fascicles within an inflammatory background); (2) The essential presence of diffuse and strong EBER positivity within the tumor cells, which is a defining feature of this entity; and (3) The systematic exclusion of a wide spectrum of other spindle cell neoplasms and inflammatory lesions through extensive immunohistochemical and molecular testing, including a negative TCR gene rearrangement which helped exclude T-cell lymphomas. The comprehensive differential diagnosis is presented in **Table 3**.

**Table 3:** Comprehensive differential diagnosis of testicular masses.

Diagnostic entity	Key positive IHC markers	Present case results and rationale for exclusion	Conclusion
Germ cell tumors	PLAP, SALL4, OCT4,CD117	All negative	Effectively Ruled out
Malignant melanoma	SOX10,HMB45, S-100	All negative	Ruled out
NK/T-cell lymphoma	TIA-1, GranzymeB, CD56	All negative; Supportive: Negative TCR gene rearrangement	Ruled out
Anaplastic large cell lymphoma	ALK	Negative	Ruled out
Inflammatory myofibroblastic tumor	ALK, Calponin	All negative	Ruled out
Langerhans cell histiocytosis	CD1α,CD68,CD163, S-100	All negative	Ruled out
Smooth muscle sarcoma	SMA,Desmin	Both negative	Ruled out
Rhabdomyosarcoma	Desmin, SMA	Both negative	Ruled out
Sarcomatoidcarcinoma	CK(Pan),EMA	Both negative	Ruled out
Classical Inflammatory Pseudotumor	EBER(typically negative)	EBER positive in tumor cells	Ruled out
EBV+ iFDCS (Present Case)	Essential: EBER (diffuse) Supportive: FDC markers (e.g., CD21, CD23, CD35)	Positive: EBER, CD35 (focal) Negative: CD21, CD23, D2-40 Supportive Negative Result: Negative TCR gene rearrangement	Definitively diagnosed

### 3.3 Treatment and prognosis

Currently, molecular research on FDCS is very limited. Some literature reports that classical FDCS may exhibit immunoglobulin gene rearrangement, while Korean scholars have reported that six cases of EBV+ iFDCS in the spleen all showed significant IgG4-positive plasma cell infiltration (15). Therefore, immunoglobulin gene rearrangement detection may be related to IgG4-positive plasma cell infiltration, but further research is needed to determine under what circumstances immunoglobulin gene rearrangement occurs in follicular dendritic cell sarcoma. Recently, scholars Bruehl et al. (16) performed next-generation sequencing on EBV+ iFDCS occurring in the spleen and intestines but did not detect any potential or clinically significant mutations or fusion genes.

The patient was discharged after an uneventful postoperative recovery and was transferred to a tertiary hospital for further management. Due to personal health considerations and other multifaceted reasons, the patient declined long-term follow-up for this study. However, we confirm that he provided written informed consent for the use of his clinical and pathological data for this academic case report and publication. Consequently, long-term survival data for this case are unavailable.

Based on the existing literature, EBV+ iFDCS typically exhibits a relatively indolent biological behavior. Although local recurrences have been reported, distant metastases are rarely observed. It is noteworthy that the tumor in this case demonstrated rapid growth over 8 months, a high Ki-67 proliferation index (approximately 60%), and easily detectable mitotic figures. These features may suggest a potentially more aggressive clinical course. EBV+ iFDCS with negative CD21 and CD23 expression is exceedingly rare, and its precise clinical prognosis requires further elucidation through the accumulation of more cases.

### 3.4 Study limitations and strengths

This study has several limitations. The lack of long-term follow-up data prevents comprehensive assessment of the tumor’s biological behavior in this rare location. The absence of peripheral blood EBV PCR testing, which limits our understanding of systemic viral load, is one of the main limitations of this study. Due to technical reasons, SSTR2 testing could not be performed, which is another limitation of this study.

However, this report has significant strengths, including being the first detailed description of primary testicular EBV+iFDCS, providing comprehensive clinical, radiological, pathological, and immunohistochemical characterization. Our findings highlight the diagnostic importance of EBER testing when FDC markers show atypical expression patterns, contributing valuable insights to the literature on this rare entity.

## 5 Conclusion

This case represents the first reported occurrence of EBV+ iFDCS in the testes. The diagnosis was confirmed through a combination of histopathological examination, immunohistochemistry, and molecular testing. Given the rarity of this tumor and its potential for misdiagnosis, a thorough understanding of its histological and immunophenotypic characteristics is essential for accurate diagnosis and management.

## Data Availability

The original contributions presented in the study are included in the article/supplementary material. Further inquiries can be directed to the corresponding author.
